# Co-teaching in medicine and nursing in training nurse anesthetists: a before-and-after controlled study

**DOI:** 10.1186/s12909-023-04827-8

**Published:** 2023-11-12

**Authors:** Xiaobei Ma, Yi Duan, Yanli Ma, Zhifeng Gao, Huan Zhang

**Affiliations:** Department of Anesthesiology, Beijing Tsinghua Changgung Hospital, School of Clinical Medicine, Tsinghua University, No. 168 Litang Road, Beijing, 102218 China

**Keywords:** Co-teaching, Medicine, Nursing, Clinical teaching, Nurse anesthetist, Nursing education

## Abstract

**Background:**

Clarifying the effectiveness of co-teaching in medicine and nursing (CMN) is important as it is crucial in clinical practice to improve the quality of patient care and prognosis. In this study, we aimed to determine the efficacy of CMN in nurse anesthetist training.

**Method:**

The study comprised a 6-month training session and a before-and-after controlled study. In total, 59 nurses were recruited. The first 30 nurses were enrolled in the conventional single-teaching in nursing (SN) group and only took nursing-related courses. The next 29 students were enrolled in the CMN group and received both general medical and nursing-specific curricula. Before and after training, medical and nursing collaboration competency scores and knowledge scores were compared between the two groups. At the end of the study, qualitative comments on teaching satisfaction and clinical reasoning skills improvement were queried, and content analysis was performed.

**Results:**

Participants in the CMN group outperformed those in the SN group in tests of medical and nursing collaboration abilities as well as knowledge. The CMN group outperformed the SN group in terms of teaching satisfaction evaluation, particularly in terms of fostering learning in the anesthetist specialty, improving clinical practice, fostering motivation, and influencing how people think about challenges at work. Furthermore, participants in the CMN group felt that their clinical reasoning abilities had improved.

**Conclusion:**

In comparison to the SN group, the CMN group had enhanced outcomes of patient care, medical and nursing collaboration, and clinical reasoning skills.

**Supplementary Information:**

The online version contains supplementary material available at 10.1186/s12909-023-04827-8.

## Background

As a collaborator with anesthesiologists [1], nurse anesthetists (NAs) have an increasingly important role in anesthesia practice, including caring for patients under anesthesia in and outside the operating room [2, 3]. Due to the field's late establishment and significant talent scarcity, on-the-job training has replaced formal education as the main way of educating NAs in China [4]. Following graduation, clinical medical units typically plan and carry out anesthetic nurse education on their own, with improvement of job competency as the primary objective [5, 6].

The development of competencies in NAs has not been fully realized, however, [7, 8] as a result of the current education of NAs, which continues to emphasize the training of basic nursing competence [9] and lacks training in medical and nursing collaborative competence as well as clinical dialectical thinking [10]. Possible causes of the problem include the following. There is a lack of uniform teaching content and teaching resources for NAs in China. There is a lack of specialized faculty to train NAs in clinical practice, and they are not experienced enough in training NAs.

Therefore, a single type of nursing education is no longer adequate to fulfill the demands of modern competencies and is not suited to the long-term development of NAs. It is believed that interprofessional education can address the aforementioned issues and enhance trainees' interprofessional synergy [11]. Interprofessional education outperforms single-mode professional instruction, according to research by Chen and colleagues [12], in terms of boosting the performance of nursing personnel and medical students in teamwork and medical activities. Similar results were obtained by Hosseinpour et al. [13]. In their training of surgical teams in clinical activities, they discovered that nursing staff might gain more from interprofessional education.

We proposed that co-teaching and co-learning in medicine and nursing might address the issues of poor medicine–nursing collaboration and the lack of knowledge regarding clinical dialectical thinking in NAs. For medicine and nursing, there is no equivalent co-teaching program or curriculum, however. In this study, we aimed to evaluate the efficacy of a collaborative medicine and nursing (CMN) teaching program as compared to a nursing program ( developed to enhance “specialist care ability” and “clinical reasoning ability” for NAs ). The teaching program included reorganization of learning materials and the course textbook, co-leaning with medical students to help NAs to better understand information and feel more confident in correctly applying procedures in complex clinical situations, as well as enhance their capacity to manage clinical problems effectively.

## Methods

### Design

**Fig. 1:**
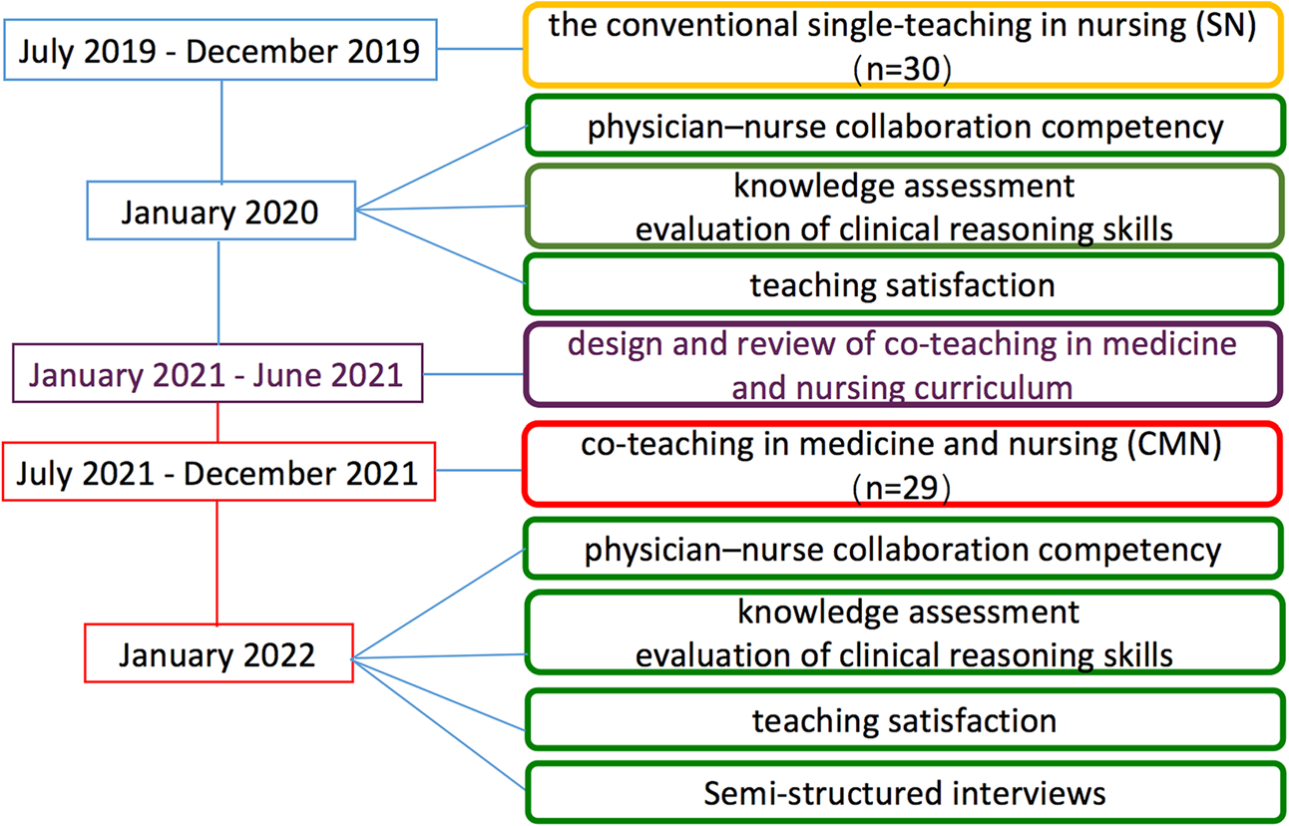
Study framework

In this historical before-and-after controlled study, we conducted an evaluation from July 2019 to December 2021 of nursing staff who had specialized training as an anesthetist in the Department of Anesthesiology, Beijing Tsinghua Changgung Hospital, China. The study framework is displayed in Fig. 1. The study has been granted an exemption from requiring ethics approval by the Ethics Committee of Beijing Tsinghua Changgung Hospital (No. 23012–6-01). All study participants signed an informed consent form after receiving an explanation of the study goal and procedure and were not compensated for it.

### Participants

A convenience sample of 59 NA students were included in this study. Inclusion criteria were as follows: the Nurse Licensure Examination has been passed by all nurses; the Nurse Licensure Examination is reliable and impartial for determining whether a candidate possesses the skills required to practice nursing; and they were voluntarily provided written consent. Exclusion criteria were as follows: nurses who dropped out of the training. Currently, although anesthesia nursing is developing rapidly in China, the total number of nurses is relatively small; therefore, all anesthesia nurses in our hospital who met the inclusion and exclusion criteria during the study time period were included in this study.

From July 2019 to December 2019, our unit adopted conventional single-teaching in nursing (SN), during which time 30 recruited trainees were enrolled into the SN group. Co-teaching in medicine and nursing (CMN) was used from July 2021 to December 2021. The CMN group was formed by the 29 trainees who were recruited during this time.

### Curriculum development for medicine and nursing

To implement CMN, we further developed a common medical and nursing course together with anesthesiologists, in addition to the nursing-specific training course with senior NAs as instructors. The curriculum design was reviewed by a panel of experts consisting of two physicians, two nurses, and one teaching professional, all with at least 5 years of teaching experience.

We matched the post competency of NAs from the International Federation of Nurse Anesthetists (IFNA) [14] with the competencies of anesthesiology residents to derive core competencies for primary care in anesthetist practice. Furthermore, we used this as the basis for curriculum screening and reorganization of the existing textbook process to obtain a common curriculum for medicine and nursing applicable to CMN (Additional file 1: Appendix 1). The content of the common curriculum ultimately included basic anesthetist theory, perioperative anesthetist management, anesthesia and analgesia management, and medical and nursing collaboration training; the curriculum was taught by teaching-qualified anesthesiologists. CMN trainees attended training together with anesthesiologist residents.

The special nursing curriculum covered basic anesthesia theory, nursing anesthesia methods for each specialty, anesthetist nursing in the post-anesthesia care unit, the management of narcotic drugs and psychotropic drugs, and a discussion of clinical nursing safety events. The teaching materials used included Clinical Anesthesiology, Morgan & Mikhail's Clinical Anesthesiology, and Nursing–Anesthesia. The curriculum was taught by senior NAs and included nursing staff only.

### Teaching methods and arrangement

The common curriculum for medicine and nursing and the nursing special curriculum together formed a structured co-teaching curriculum in medicine and nursing for the training of the CMN group. The SN group received the special nursing curriculum only. The common curriculum for medicine and nursing was taught every Thursday for 45 min.

In the co-teaching in medicine and nursing, anesthesiologists led lectures and NAs and anesthesiologist residents were trained as a group. Teaching was driven by clinical activities and oriented to post competency. Teaching methods included lectures by the instructor, case discussions, and exercises simulating clinical activities that were conducted by medical and nursing teams of NAs and anesthesiologist residents. The curriculum was aimed at improving the professional knowledge base of NAs and the capacity for physician–nurse collaboration.

The period of this training was 6 months. At the end of training, trainees were given knowledge and clinical practice assessments, and surveys and interviews were conducted on teaching satisfaction. To maintain training consistency, all faculty members received training in providing step-by-step scripted guidance. All instructors had at least 5 years of work experience and 3 years of teaching experience. Figure 1 depicts the study timeline.

### Indicators

#### Key outcome indicators

##### The key outcome indicator was physician–nurse collaboration competency scores

Before the start of training and after the end of training, the assessment team selected eligible clinical cases and used the Objective Structured Clinical Examination (OSCE) to conduct the assessment. The examination was conducted with the cooperation of an attending anesthesiologist on staff. The assessment includes four stations: preoperative anesthesia preparation, induction of anesthesia, anesthesia management, and post-anesthesia recovery care. The assessment is based on two dimensions: anesthesia task performance and safe patient care, with a total score of 180 points. Anesthesia task performance was assessed using a tool developed for this study. The tool consists of a total of 16 items, including 5 items on skills needed to achieve patient outcomes, 5 items on the provision of patient-centred care, 3 items on attitudes need to improve team collaboration, and 3 items on professional responsibilities. The scale uses a 5-point Likert scale, ranging from 1 for “strongly disagree” to 5 for “strongly agree,” with a total of 80 points. The face validity and content validity were completed with the panel. The panel comprised 4 anesthesiologist specialists, 2 NA specialists, 9 attending physicians, and 9 NA instructors. A questionnaire was conducted among the panel to assess the face validity and content validity, including realism and utility. The questionnaire is based on a ten-point scale, ranging from 0 for “not realistic/not useful” to 10 for “realistic/useful”. face validity: The majority of the panel considered the tool to be “very realistic” (median 9/10). Content validity: the panel also approved the tool was a “very useful” evaluation tools for NAs (median 9/10). The tool was completed by the examiner who was observing the NA students during the exam. Safe patient care was assessed primarily using the operational assessment form, which was scored with a total of 100 points. The tool was also completed by the examiner who was observing the NA students during the exam.

#### Secondary outcome indicators

##### Knowledge evaluation results

Before the start of training and after the end of training, the assessment team developed tests according to the syllabus and teaching objectives and organized examinations to assess the expertise of NAs. The assessment team comprised two physicians, two nurses, and one teaching specialist. The examinations were reviewed by a panel of experts and required a reliability (reflecting the degree of consistency) > 0.85 [15], discrimination (reflecting the quality of the paper and the basis for screening the questions) at 0.2–0.3, which was conducive to distinguishing differences in students' abilities and also had good control over the failure rate [16, 17], and a medium difficulty of 0.3–0.8 [18].

##### Evaluation of clinical reasoning skills

Drawing on the Medical Student Clinical Reasoning Skills Assessment Form, trainees self-assessed whether their clinical reasoning skills had improved. The Cronbach's α coefficient of the scale was 0.91, and the test–retest reliability was 0.84. The scale includes six items on critical thinking skills, 11 on systematic thinking skills, and seven items on evidence-based thinking skills. A total of 24 items in three dimensions are used to assess the clinical reasoning skills of medical students. As for the evaluation criteria, a 5-point Likert scale was used to assign values, as follows: very good = 5 points, good = 4 points, average = 3 points, poor = 2 points, and very poor = 1 point. The full score is 120, which was converted to 100. A clinical reasoning skills score of 80–100 is considered very good, 60–80 is good, 40–60 is average, 20–40 is poor, and a clinical reasoning skills score of 0–20 is considered very poor.

##### Teaching satisfaction

A self-designed student evaluation questionnaire was used to investigate students’ evaluation of the teaching model after training. The questionnaire consisted of seven entries, including difficulty of the course; whether the training course had facilitated study of the specialty of anesthetist; whether the training course helped improve clinical workability, motivation to learn, and motivation to work; whether the training course had an impact on the manner of thinking about clinical work; and whether clinical work experience was improved. Each topic was rated using a 5-point Likert scale, ranging from 1 for “strongly disagree” to 5 for “strongly agree”. The questionnaire was designed and modifed according to previous studies and the purpose of this study, with its validity verifed in a previous study [19, 20].

##### Semi-structured interviews

At the end of training, some participants were randomly selected by the teaching staff for semi-structured interviews. The interviews addressed feelings about co-teaching in medicine and nursing, suggestions for future course content and format, and professional identity.

### Statistical analysis

Kolmogorov–Smirnov test was used to assess the normality of continuous variables. Measures satisfying a normal distribution are expressed as mean ± standard deviation, and as median (interquartile range) with a non-normal distribution. Categorical variables are reported as frequency and percentage. Comparisons between groups were made using *t*-tests for normally distributed data and Mann–Whitney U-tests for non-normally distributed data. Comparisons between groups of count data were made using the chi-square test. Repeated measures information such as pre- and post-training healthcare collaboration competency scores and knowledge evaluation scores were analyzed using repeated measures analysis of variance. The data in this study were analyzed using IBM SPSS 26.0 (IBM Corp., Armonk, NY, USA), and statistical significance was set at *P* < 0.05.

## Results

**Fig. 2:**
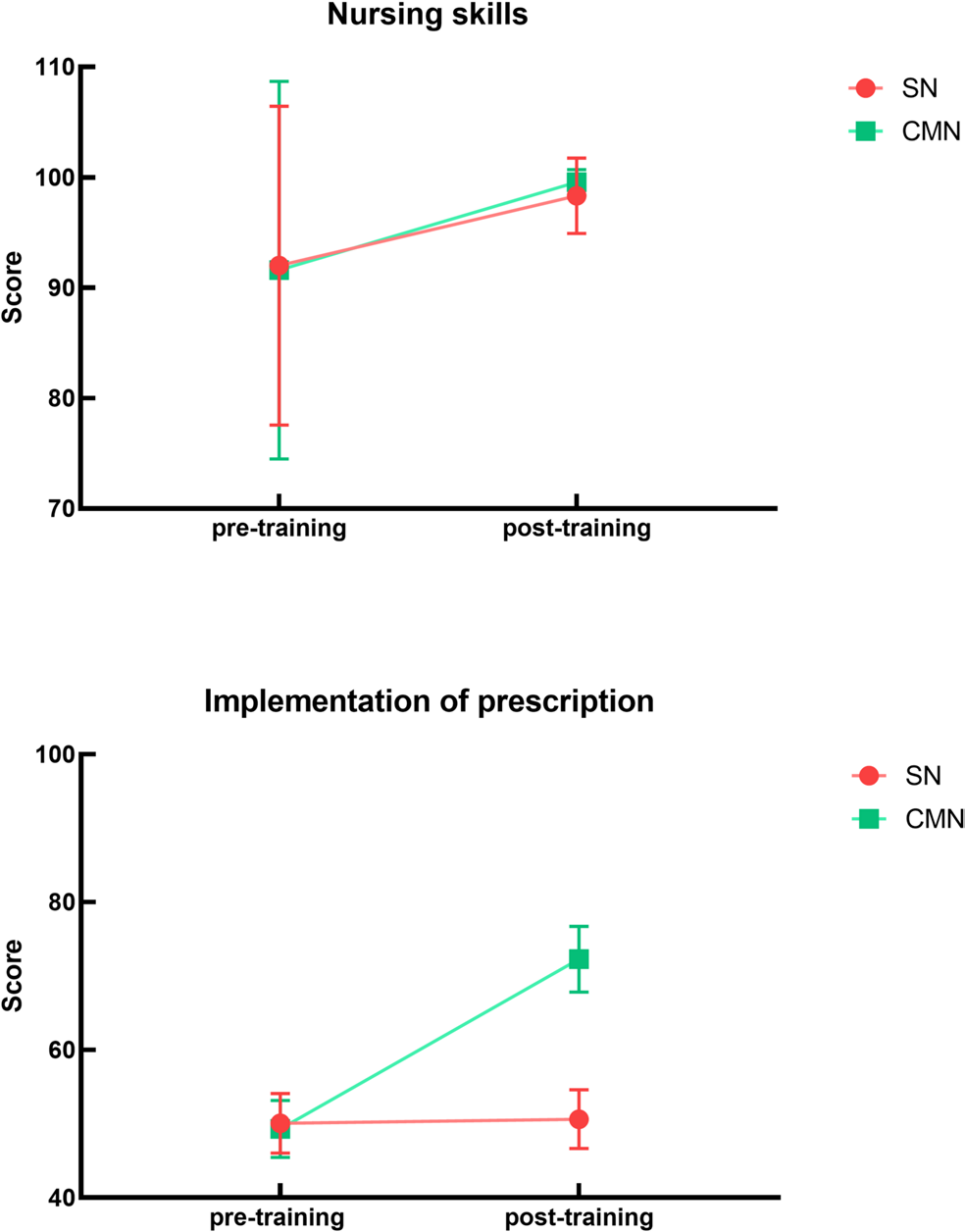
Comparison between CMN and SN in terms of the capacity for physician–nurse collaboration

A total of 59 NAs participated in this study and completed the training and assessment, 30 in the SN group and 29 in the CMN group. There were no significant differences between the two groups in terms of age, sex, and years of work experience (Table 1). Within-group comparisons showed a significant improvement in the CMN group after training in terms of capacity for physician–nurse collaboration (140.90 ± 16.83 vs. 171.83 ± 4.35, *P* < 0.01), mainly in the dimension of medical task performance (49.31 ± 3.84 vs. 72.28 ± 4.45, *P* < 0.01) (Fig. 2). We assessed the expertise of our trainees in knowledge tests. In this study, the reliability of all examinations was 0.85–0.87, the differentiation was 0.2–0.3, and the difficulty was 0.72–0.8. All examinations were of high quality. The knowledge evaluation scores in the CMN group increased from 61.84 ± 8.32 to 74.52 ± 10.30, and the difference was statistically significant (*P* < 0.01).

**Table 1:** Demographic characteristics of participants

	CMN group (*n* = 29)	SN group (*n* = 30)	t/χ2/Z	*P* value
Age (y), mean ± SD	26.38 ± 4.72	27.50 ± 4.56	− 0.927	0.358
Years of work (y)	3.67 ± 3.03	2.97 ± 2.16	1.028	0.309
Sex	/	/	/	/
Male, n (%)	3 (10.34%)	6 (20.00%)	1.063	0.302
Female (n, %)	26 (89.66%)	24 (80.00%)

**Fig. 3:**
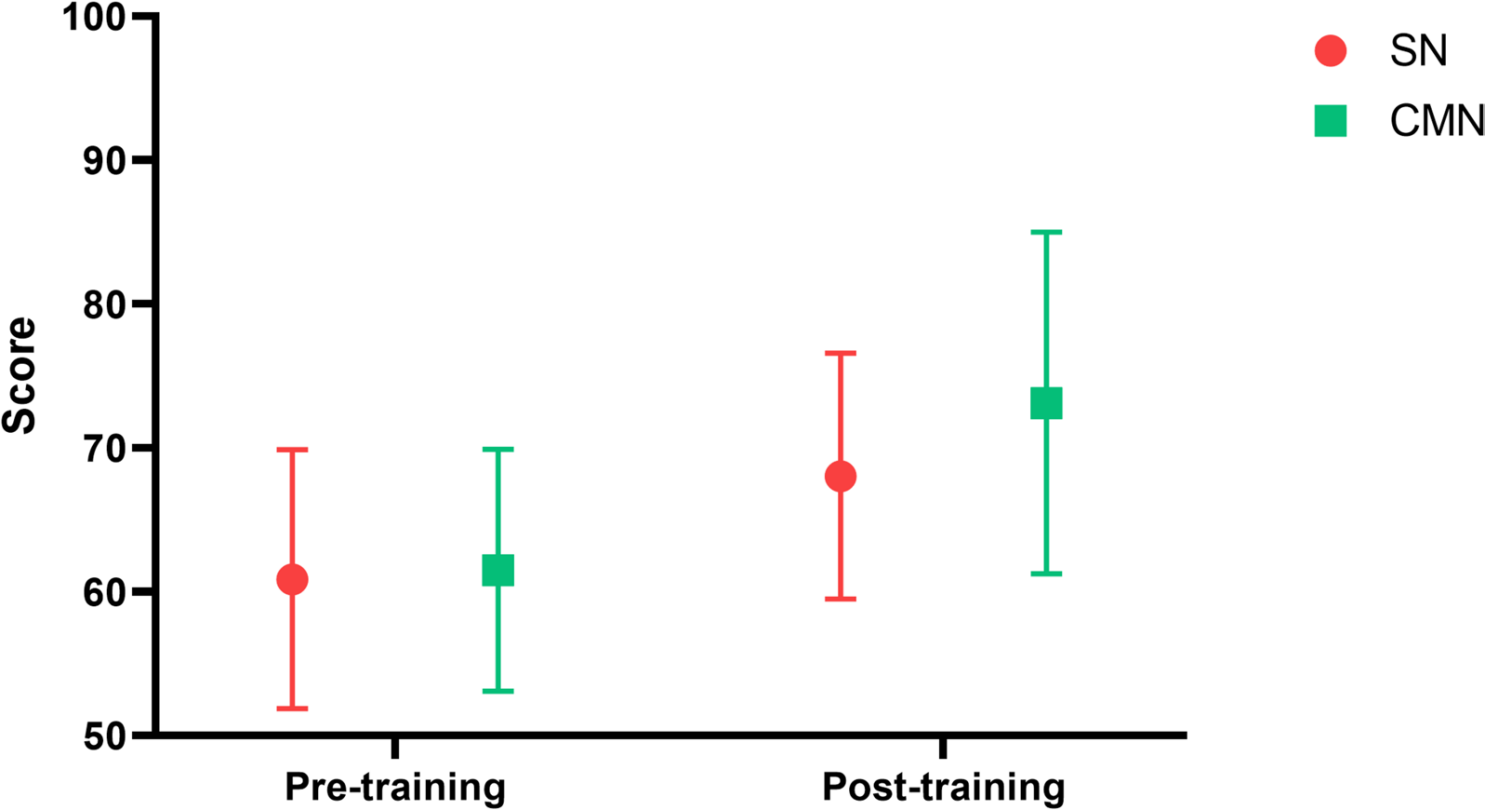
Comparison between CMN and SN in terms of knowledge evaluation

The scores of capacity for physician–nurse collaboration in the SN group were also improved compared with before training (142.03 ± 13.39 vs. 149.00 ± 4.84); however, there was no significant difference. The knowledge evaluation scores in the SN group were significantly improved after training compared with before training (60.85 ± 9.00 vs. 68.02 ± 8.55, *P* = 0.002) (Fig. 3).

Comparisons between groups showed that NAs in the CMN group had a significantly greater capacity for physician–nurse collaboration than those in the SN group after training (171.83 ± 4.35 vs. 149.00 ± 4.84, *P* < 0.01), especially in the dimension of medical task performance (72.28 ± 4.45 vs. 50.63 ± 3.97, *P* < 0.01) (Fig. 2). In terms of expert assessment, the CMN group was similarly better than the SN group; the difference was statistically significant, but the actual score difference was small and the practical significance was weak (74.52 ± 10.30 vs. 68.02 ± 8.55, *P* = 0.011) (Fig. 3).

Participants in the CMN group rated the improvement in their clinical reasoning skills owing to the co-teaching curriculum in medicine and nursing, with scores above 4 points in all dimensions. The scores were, from highest to lowest, systems thinking skills, critical thinking skills, and evidence-based thinking skills. Compared with the SN group, there was a significant difference in the improvement of clinical reasoning ability in this group (82.66 ± 4.42, *P* < 0.01). Specific scores are shown in Table 2.

**Table 2:** Nurse anesthetist evaluation of whether co-teaching curriculum in medicine and nursing improved their clinical reasoning skills

	**CMN group** **(*n* = 29)**	**SN group** **(*n* = 30)**	***t***	***P* value**
**Overall** e**valuation**	82.66 ± 4.42	70.23 ± 4.34	− 10.888	0.000
Systems thinking skills	4.45 ± 0.63	/		
Critical thinking skills	4.39 ± 0.72	/		
Evidence-based thinking skills	4.21 ± 0.75	/		

**Fig. 4:**
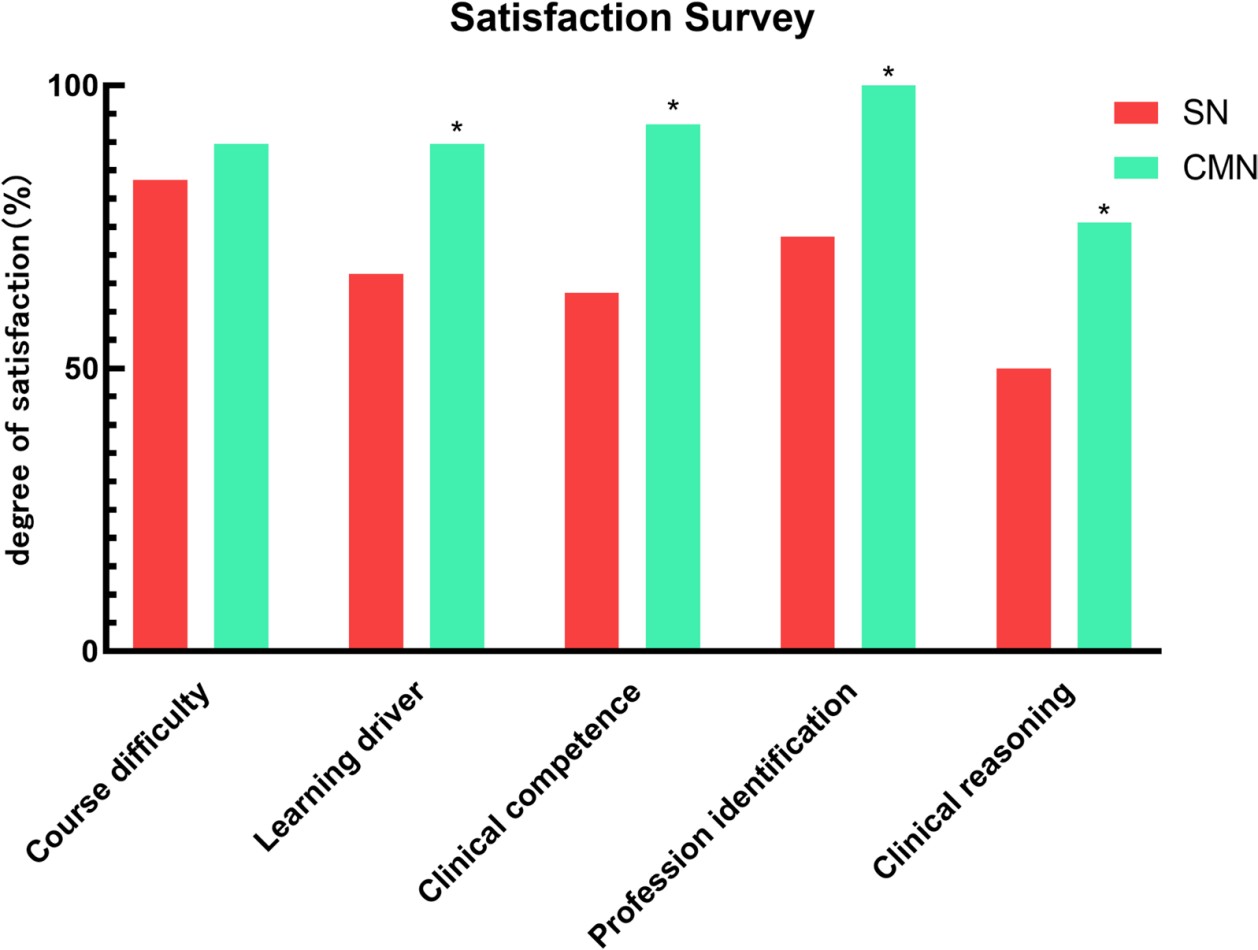
Satisfaction survey. CMN * compared with SN, *P* < 0.05

In the questionnaire to assess satisfaction with the curriculum, we categorized “generally agree,” “agree,” and “strongly agree” as “agree” and “disagree” and “strongly disagree” as “disagree.” The results showed that both groups of participants were very positive about post-graduate training. The CMN group felt that co-teaching was helpful in improving clinical competence (63.3% vs. 93.1%, *P* < 0.05) and clinical reasoning (50% vs. 75.8%, *P* < 0.05), and was more helpful in enhancing learning driver (66.7% vs. 89.6%, *P* < 0.05) and professional identity (73.3% vs. 100%, *P* < 0.05) (Fig. 4).

We conducted semi-structured interviews with a random sample of eight participants from the CMN group, all of whom expressed satisfaction with the training approach. These NAs considered that the course: (1) improved collaboration between physicians and nurses. “ In the course of the lecture, I will link the medical knowledge with the clinical problems encountered.”; “ I usually pay more attention to nursing issues and write down the relevant medical priorities after listening to the lectures, which also helps me to communicate with physicians.”, (2) promoted their knowledge and understanding of anesthesia care and medical treatment. “ Some content cannot be addressed in the nursing classroom, but the answers can be found here.”; and (3) was useful for clinical work. “ So many things that I can't understand I suddenly figured out.” However, the NAs felt that the relevance, hierarchy, and lecture style could be improved as follows. (1) For nursing staff with zero foundation, it was difficult to understand the basic knowledge involved in the common course, and it should be explained in greater detail. (2) During the lecture, the teacher should set the focus of the lecture more clearly. (3) More clinical cases can be combined with the explanation of basic knowledge points. (4) The introduction of surgery-related aspects might be increased. Suggestions for faculty were as follows. (1) The instructors had rich professional knowledge and clinical experience, but in the process of knowledge transfer, they did not transmit information sufficiently well, and the lectures lacked interest and clarity. (2) Some nursing instructors did not have a clear hierarchy in their lectures, the focus was not very clear, and nursing problems were not clearly explained, resulting in a lack of systematic understanding of the specialty.

## Discussion

In this study we evaluated a collaborative medical and nursing curriculum (CMN) as compared to a single teaching in nursing curriculum (SN) on knowledge regarding the safe and patient centred delivery of anesthesia care by NA. The results of the study showed that post-graduate clinical teaching of NAs, using either single-mode nursing education or a co-teaching curriculum in medicine and nursing, was beneficial in improving professionalism and clinical care among NAs. The IFNA states that post competency roles for NAs should include Professional, Communicator, Collaborator, Manager, Health Advocate, and Scholar. The strength of this study lay in the development and design of an interprofessional teaching curriculum with the collaborative participation of physicians and nurses, using the post competency of NAs as a framework, to strengthen the training of medical and nursing collaboration skills and clinical reasoning and help participants to meet the current competency requirements of NAs.

Our findings showed that co-teaching curricula in medicine and nursing had greater advantages in improving NAs’ capacity for physician–nurse collaboration, compared with single-mode nursing instruction. Traditionally, nurses and physicians have had different educational programs. Nurses are trained to focus on overall health and quality of life whereas physicians are trained to diagnose and treat disease. Although nurses and physicians are educated separately, how effectively nurses and physicians work together to care for patients is critical in clinical work. Hospital administrators should require hospitals to provide hospital-based interprofessional education and practice programs for nurses and physicians, and these programs should be designed such that nurses and physicians learn from each other [21].

The establishment of a co-teaching curriculum in medicine and nursing in this study meets the effective cooperation between nurses and physicians. Many studies have found that nurses are very willing to collaborate with physicians [22]. During the interviews with NAs in our study, many expressed the following thoughts. “In the course of the lecture, I will link the medical knowledge with the clinical problems encountered.” “I usually pay more attention to nursing issues and write down the relevant medical priorities after listening to the lectures, which also helps me to communicate with physicians.” “So many things that I can't understand I suddenly figured out.” “Some content cannot be addressed in the nursing classroom, but the answers can be found here.” Thus, cooperative teaching between physicians and nurses helped promote cross-fertilization of different teaching content, which expanded the depth and breadth of anesthetist nursing education, improved students' motivation for learning, and enhanced their clinical work experience. Therefore, to a certain extent, the co-teaching curriculum in medicine and nursing is an effective way for anesthesiologists and nurses to interchange information, which has guiding importance for clinical practice.

In participants' self-assessments, we also found that the co-teaching curriculum in medicine and nursing helped to develop clinical dialectical thinking. Clinical reasoning skills refer to the ability of nurses to analyze, reason logically, make clinical judgments, and make decisions about the diagnosis and treatment of diseases to solve problems in clinical practice [23]. In clinical practice, nurses are required to be able to correctly apply nursing procedures in complex clinical scenarios, to propose solutions to different problems that exist at each stage of the patient's care, and to evaluate their effectiveness [24]. Clinical reasoning skills are important competencies that clinical nurses must have, and good clinical reasoning skills can improve overall clinical competence [25]. Critical clinical reasoning is a necessary skill for clinical caregivers who can help them analyze clinical situations to make quick and correct decisions [26]. Critical clinical reasoning is necessary if professional nurses wish to provide high-quality care [27, 28]. Researchers have found that most professional nurses have low to moderate levels of critical thinking skills [29, 30]. Novice nurses or recent nursing graduates have a difficult time making appropriate clinical decisions in an extremely complex, fast-paced, healthcare environment. With limited time and resources, it is important to enhance the development of higher-level clinical reasoning skills among novice registered nurses [31]. Studies in China have also shown that nursing staff have poor critical and evidence-based thinking and are not skilled at identifying and solving clinical problems [32, 33]. More than half of new nurses fail to promptly identify clinical problems and make prudent clinical decisions using multiple problem-solving approaches [33]. The clinical reasoning skills of senior nurses are improved to some extent, but some senior nurses still make decisions based on their prior work experience [32].

Finally, although the co-teaching curriculum in medicine and nursing took a great deal of time and energy according to trainees, it was popular among participants, generating high levels of satisfaction given its advantages in improving medical and nursing collaboration and enhancing professional competence.

## Limitations

The focus of this study was on the expansion of knowledge and the improvement of clinical reasoning skills, with fewer interventions for the training of NAs in technical operations. In the future, there is a need to explore additional collaborative teaching models between physicians and nurses and to innovate skills training methods that are suitable for NAs, to enhance the operational ability of NAs in China. In the curriculum of this study, few courses were focused on improving evidence-based thinking skills. However, given the job content and work nature of NAs, this part is indispensable and should be appropriately adjusted in future teaching processes. Furthermore, this study was historically controlled; the participants were different in different periods, so there were individual differences, the teaching situation in the first and second stages was different, and there were different biases and confounding factors. Future studies will be conducted to examine the effectiveness of the collaborative medical and nursing teaching model in NA education in other parts of the country. There is also an opportunity to evaluate if the medical students who participate in this joint teaching model learn how to collaborate more effectively.

## Conclusions

In summary, we used a co-teaching curriculum in medicine and nursing, guided by job competency, to effectively improve clinical collaboration and professional competence among NAs by reconstructing the faculty and reorganizing the textbook process. This teaching model served as a positive exploration of post-graduate education and training of NAs in hospitals and had good implications for the training of NAs in China.

### Supplementary Information

**Additional file 1:** **Appendix 1.** The general curriculum of co-teaching in medicine and nursing.

## Data Availability

The datasets used and/or analyzed during the current study are available from the corresponding author on reasonable request.
