# Supplementary material for: Co-teaching in medicine and nursing in training nurse anesthetists: a before-and-after controlled study
Source: BMC Med Educ. 2023 Nov 12;23:856. doi: 10.1186/s12909-023-04827-8 (PMC10641995; doi:10.1186/s12909-023-04827-8)
Supplement: Supplementary file 1 — Additional file 1: Appendix 1. The general curriculum of co-teaching in medicine and nursing. [file 12909_2023_4827_MOESM1_ESM.docx]

Appendix 1 The general curriculum of co-teaching in medicine and nursing

| **No.** | **Topic** | **Lecturers** | **Teaching time** |
| --- | --- | --- | --- |
| **1** | Hemodynamic monitoring and clinical significance | Attending and above anesthesiologists | Thursdays 7:20-7:50 |
| **2** | Anesthesia and cerebral blood flow, cerebral metabolism |  |  |
| **3** | Anesthesia and Respiration |  |  |
| **4** | Anesthesia and Circulation |  |  |
| **5** | Anesthesia and Blood |  |  |
| **6** | Anesthesia and the Kidneys |  |  |
| **7** | Anesthesia and the liver |  |  |
| **8** | Anesthesia and Endocrinology |  |  |
| **9** | Anesthesia and Stres |  |  |
| **10** | Water-electrolyte balance and imbalance |  |  |
| **11** | Acid-base balance and imbalance |  |  |
| **12** | Myorelaxants and myorelaxation monitoring and antagonism |  |  |
| **13** | Drugs acting on adrenergic receptors |  |  |
| **14** | Cholinomimetic and anticholinergic drugs |  |  |
| **15** | Vasodilators and cardiotonic drugs |  |  |
| **16** | Tracheal intubation and lung isolation |  |  |
| **17** | Respiratory management during anesthesia |  |  |
| **18** | Circulatory management during anesthesia |  |  |
| **19** | Serious complications during general anesthesia |  |  |
| **20** | Hypothermia and controlled hypotension |  |  |
| **21** | Complications in the anesthesia recovery room and during the awakening period |  |  |
| **22** | Anesthesia for day surgery |  |  |
| **23** | Principles of sedation and analgesia for non-anesthetized patient |  |  |
| **24** | Postoperative nausea and vomiting prevention guidelines |  |  |
| **25** | Principles of management of postoperative analgesia |  |  |
| **26** | Anesthesia for non-cardiac surgery in cardiac patients |  |  |
| **27** | Pediatric Anesthesia |  |  |
